# Neglecting Colds

**Published:** 1860-04

**Authors:** 


### NEGLECTING COLDS.

EVERY intelligent physician knows that the best possible method of promptly curing a cold is, that the very day in which it is observed to have been taken, the patient should cease absolutely from eating a particle for twenty-four or forty-eight hours, and should be strictly confined to a warm room, or be covered up well in bed, taking freely hot drinks. It is also in the experience of every observant person, that when a cold is once taken, very slight causes indeed increase it. The expression, "It is nothing but a cold," conveys a practical falsity of the most pernicious character, because an experienced medical practitioner feels that it is impossible to tell in any given case, where a cold will end; hence, and when highly valuable lives are at stake, his solicitudes appear sometimes to others to verge on folly or ignorance. A striking and most instructive example of these statements is found in the case of Nicholas the First, the Emperor of all the Russias. For more than a year before his death, his confidential medical adviser observed that in consequence of the Emperor "not giving to sleep the hours needed for restoration," his general vigor was declining, and that exposures which he had often encountered with impunity, were making unfavorable impressions on the system—that he had less power of resistance. At length, while reviewing his troops on a January day, he took a severe cold, which at once excited the apprehensions of his watchful physician, who advised him not to repeat his review.

"Would you make as much of my illness if I were a common soldier?" asked the Emperor, in a tone of good-natured pleasantry.

"Certainly, please your majesty; we should not allow a common soldier to leave the hospital if he were in the state in which your majesty is."

"Well, you would do your duty—I will do mine," and the exposure was repeated, with the result of greatly increasing the bad effects of this original cold, and he died in a week afterwards.

It is not the weakness of a few great men to transfer their superiority in other things to their knowledge of health and medicine. The self-reliant or self-opinionated have been often heard to exclaim: "I believe I know about as much as the doctors. A doctor don't know more than any body else." One of the most eminent clergymen of his sect recently died, learned above any of his fellows, could write and converse in some half-a-dozen languages. An intimate friend and panegyrist said of him, that he held medical science in a kind of contempt, had little or no confidence in medicines or physicians. These are not the exact words, but they embody the impression which the exact words would make on ordinary minds. The result was, that he kept ailments to himself for more than a year; ailments whose nature is to go on steadily and become more and more aggravated to a fatal issue; but which judicious remedial means have a thousand times eradicated. He died in the very prime of intellectual manhood.

The pilot, who has a thousand souls aboard, is many a time almost crazed with a sense of his responsibility, when he is steering his vessel over dangerous places, while the passengers themselves see nothing but unrippled waters and the clear blue sky; at the same time, a quarter's turn of the wheel, more or less, would, in the briefest space, send them all unannealed and unshriven into the presence of their Maker. Hence, in a well-regulated ship, a passenger is never allowed to address a word to the man who is at the wheel. Thus it is with an intelligent physician in reference to his patient, and he is wise who will read the lesson well by remembering that it is his business to do and not to babble; for the people's ignorance of nature and her operations, as to the human body, is amazing to those whose stock of amazement has not long ago been utterly exhausted in the contemplation of the stupidities of mankind.

---

**Health Tracts** of one page are sold and sent for 25 cents a hundred, by addressing Dr. W. W. Hall, New-York. Subjects: Winter Shoes, Wearing Flannel, Care of the Eyes, Cold Feet, Regulating the Bowels, How to Sit, Walk, Breathe, Curing Colds, Avoiding Disease, Best Hair Wash, etc.

